# Global Carrier Rates of Rare Inherited Disorders Using Population Exome Sequences

**DOI:** 10.1371/journal.pone.0155552

**Published:** 2016-05-24

**Authors:** Kohei Fujikura

**Affiliations:** Kobe University School of Medicine, 7-5-1, Kusunoki-cho, Chuo-ku, Kobe, 650-0017, Japan; Odense University Hospital, DENMARK

## Abstract

Exome sequencing has revealed the causative mutations behind numerous rare, inherited disorders, but it is challenging to find reliable epidemiological values for rare disorders. Here, I provide a genetic epidemiology method to identify the causative mutations behind rare, inherited disorders using two population exome sequences (1000 Genomes and NHLBI). I created global maps of carrier rate distribution for 18 recessive disorders in 16 diverse ethnic populations. Out of a total of 161 mutations associated with 18 recessive disorders, I detected 24 mutations in either or both exome studies. The genetic mapping revealed strong international spatial heterogeneities in the carrier patterns of the inherited disorders. I next validated this methodology by statistically evaluating the carrier rate of one well-understood disorder, sickle cell anemia (SCA). The population exome-based epidemiology of SCA [African (allele frequency (AF) = 0.0454, N = 2447), Asian (AF = 0, N = 286), European (AF = 0.000214, N = 4677), and Hispanic (AF = 0.0111, N = 362)] was not significantly different from that obtained from a clinical prevalence survey. A pair-wise proportion test revealed no significant differences between the two exome projects in terms of AF (46/48 cases; *P* > 0.05). I conclude that population exome-based carrier rates can form the foundation for a prospectively maintained database of use to clinical geneticists. Similar modeling methods can be applied to many inherited disorders.

## Introduction

Recent advances in next-generation sequencing (NGS) technology have revolutionized the field of clinical genetics [1–4]. This technology has facilitated the identification of the novel causative genes for >3,000 inherited disorders, which are currently annotated in the Online Mendelian Inheritance in Man (OMIM) [2, 3]. Most of these disorders are referred to as rare or orphan diseases because of their low incidence [5]. In clinical practice, molecular genetic testing is already being applied to screen for these inherited disorders [6]. However, the epidemiological information of many inherited disorders is completely insufficient and inconclusive. Particularly for rare diseases, epidemiology is a research field that remains largely unexplored by clinical geneticists and researchers [7]. Total global prevalence of all monogenic disorders at birth has been calculated to be several percent [5]. In Canada, it has been estimated that single-gene disorders may account for approximately 40 percent of cases in pediatric practice [8]. Therefore, the public health impact of Mendelian diseases is a topic of growing interest worldwide. Reliable estimates of the populations affected by inherited diseases have become increasingly important to guide efficient allocation of public health resources in each country, region, and city [7, 9, 10].

The lack of epidemiologic studies of inherited disorders is particularly true for developing countries with limited resources [11–13]. Most epidemiologic researches have been conducted with individuals from Europe and North America, who represent only a fraction of the global population [11, 12]. In developing countries, consultation rates, data collection methods, and population-based registries for inherited disorders vary considerably by urbanization grade and ambient environment [11–13].

To overcome these limitations I analyzed the global carrier rates of rare inherited disorders using geographical population exomes. The global map of the carrier rates showed strong population-specificity and this prediction represented equivalent accuracy that may be achievable with clinical practice. This is an initial global overview of the carrier rate of genetic disorders using population exome sequences.

## Results

### Strategy for epidemiological research on Mendelian disorders using population exome sequences

**Fig 1:**
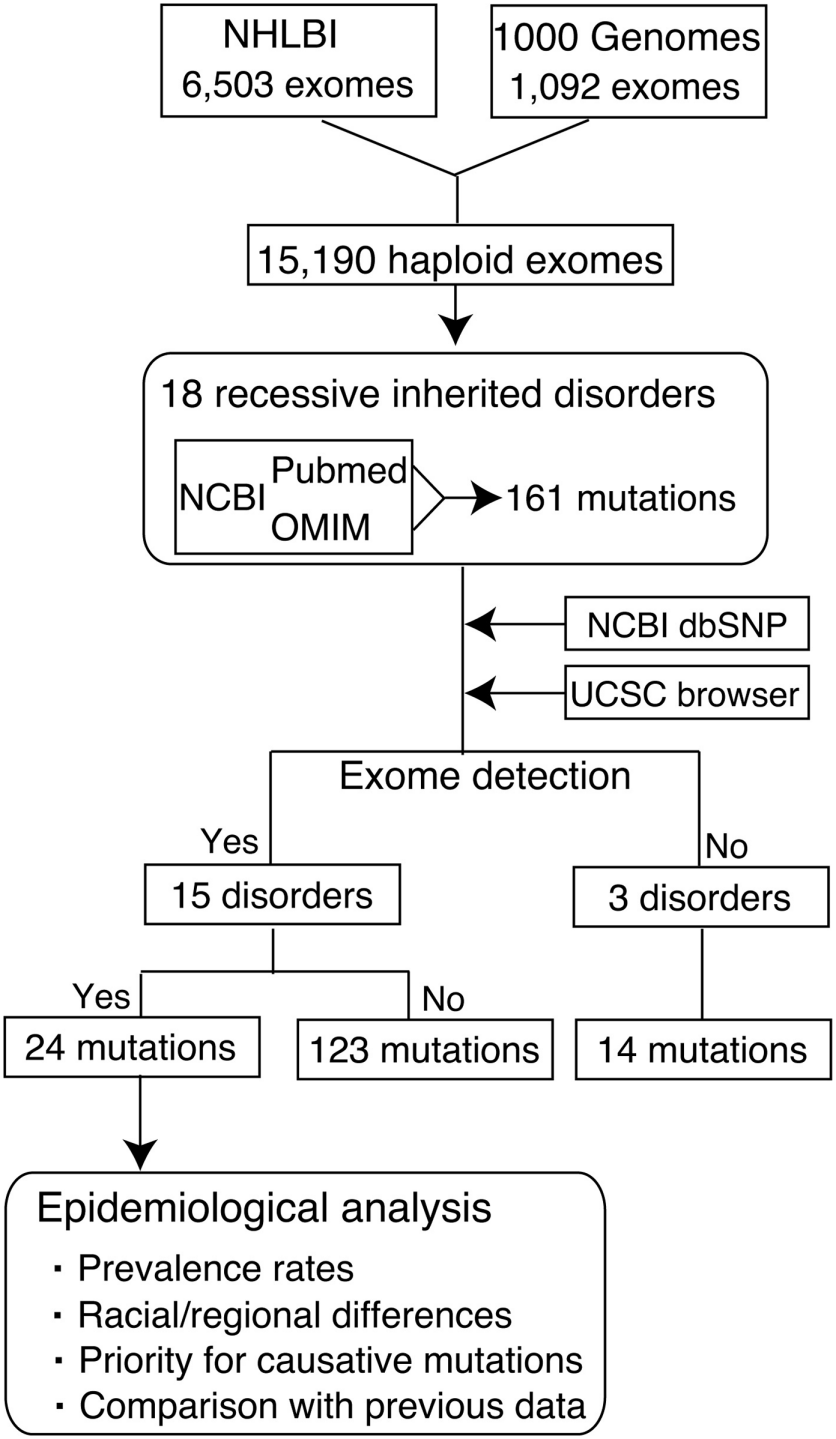
Strategy for epidemiological research on Mendelian disorder using exome sequences. A flow chart used to study the geographic prevalence shows the process of mutation detection using 1000G and NHLBI datasets. A total of 15,190 haploid exomes were screened for 161 causative mutations linked to 18 genetic disorders. Several platforms (NCBI dbSNP and UCSC Browser) were used to access the validity of mutations and examine previous information on gene annotations and alleles.

As an initial study toward determining the genetic epidemiology of inherited disorders, genetic pipelines from 1000 Genomes (1000G) [14, 15] and National Heart, Lung, and Blood Institute (NHLBI) projects [16, 17] were collected for variations with the potential to affect protein integrity (Fig 1). The dataset included the exome and its surrounding intronic sequences for 1,092 individuals (525 males, 567 females) of 14 ethnic origins and 6,503 individuals (2,443 males and 4,060 females) of two ethnic origins. Population demographics are summarized in S1 Table. Caucasians comprised 34.7% and 66.1% of subjects from the 1000G and NHLBI groups, respectively. Asian and Hispanic populations, which were represented only in the 1000G, constituted 26.2% and 16.6% of the group, respectively. A total of 65.9% were female. Many samples were from within the United States; a minority were from China, Japan, Colombia, Mexico, Puerto Rico, Finland, England, Spain, Germany, Italia, Nigeria and Kenya. These populations under the study are likely depleted for individuals with rare genetic disorders, but when the prevalence rates are so close to 0 (<0.25%) under Hardy-Weinberg equilibrium the carrier rate is usually approximated as follows:

$$p^{2}+2pq\times 0.5=p^{2}+pq\approx pq$$ (1)

where *p* and *q* indicates allele frequencies and *p* + *q* = 1 (*p*<0.05; *q*>0.95).

Disease panels used in this initial study were as follows: Sickle cell anemia (SCA; OMIM #603903); Primary immunodeficiency (Mucocutaneous fungal infection) (#613108); Pituitary hormone deficiency, combined 2 (CPHD2; #262600); Canavan disease (#271900); Pustular psoriasis (No description in OMIM); Rod-cone dystrophy (RCD; #615780); Primary autosomal recessive microcephaly 1 (MCPH1; #251200); Seckel syndrome 5 (SCKL5; #613823); Pontocerebellar hypoplasia type 1B (PCH1B; #614678); Miller syndrome (#263750); Facial dysmorphism, lens dislocation, anterior-segment abnormalities, and spontaneous filtering blebs (FDLAB, or Traboulsi syndrome; #601552); Carpenter syndrome 1 (CRPT1; #201000); Glucocorticoid deficiency 4 (GCCD4; #614736); Childhood-onset dilated cardiomyopathy (#615916); Usher syndrome type 1J (USH1J; #614869); Aicardi-Goutières syndrome 6 (AGS6; #615010); 3-methylglutaconic aciduria with deafness, encephalopathy, and Leigh-like syndrome (MEGDEL syndrome; #614739); and Severe dermatitis, multiple allergies and metabolic wasting syndrome (SAM syndrome; #615508) (S2 Table). The list of mutations was manually collated from all literature sources published over a wide period (from 1957 to 2014) (See S2 Table). In addition, several gene/disease annotation systems, including NCBI Entrez and OMIM, were used to identify disease-causing mutations (Fig 1). I identified a total of 161 mutations associated with 18 recessive diseases (S2 Table), of which 24 mutations were detected in both or either of the two exome datasets (Table 1). 15 genetic diseases were detected in a total of 7,595 individuals while three disorders, childhood-onset dilated cardiomyopathy, USH1J, and SAM syndrome, were not (Fig 1). Causative alleles were classified by mutation type, carrier rate, racial group, and clinical impact (Fig 1 and Table 1).

**Table 1:** Estimated carrier rates of 15 Mendelian disorders by race, ethnicity, and country. The information about the mutation and carrier rate is shown in this figure. Pustular psoriasis caused by is yet described in OMIM. The abbreviations are as follows: AA, African Americans; EA, European Americans; ASW, American’s of African Ancestry in SW; CEU, Utah Residents (CEPH) with Northern and Western European ancestry; CHB, Han Chinese in Beijing; CHS, Southern Han Chinese; CLM, Colombian from Medellin; FIN, Finnish in Finland; GBR, British in England; IBS, Iberian population in Spain; JPT, Japanese in Tokyo; LWK, Luhya in Webuye; MXL, Mexican ancestry from Los Angeles; PUR, Puerto Rico from Puerto Rica; TSI, Toscani in Italia; YRI, Yoruba in Ibadan.

	1	2	3	4		5				6	7	8
Disease name	Sickle cell anemia (SCA)	Primary immunodeficiency (Mucocutaneous fungal infection)	Pituitary hormone deficiency, combined 2 (CPHD2)	Canavan disease		Pustular psoriasis				Rod-cone dystrophy (RCD)	Primary autosomal recessive microcephaly 1 (MCPH1)	Seckel syndrome 5 (SCKL5)
OMIM entry	#603903	#613108	#262600	#271900		No description				#615780	#251200	#613823
Gene name	*HBB*	*CLEC7A*	*PROP1*	*ASPA*		*AP1S3*				*PLK1S1*	*MCPH1*	*CEP152*
RefSeqGene	NG_000007.3	NG_016291.1	NG_015889.1	NG_008399.1		NG_034017.1				NG_033122.1	NG_016619.1	NG_027518.1
mRNA ID	NM_000518.4	NM_197947.2	NM_006261.4	NM_000049.2		NM_001039569.1				NM_018474.4	NM_024596.3	NM_001194998.1
Mutation	c.20A>T (p.Glu7Val)	c.714T>G (p.Tyr238*)	c.301_302delAG (p.Leu102Cysfs*8)	c.854A>C (p.Glu285Ala)	c.914C>A (p.Ala305Glu)	c.11T>G (p.Phe4Cys)	c.64A>G (p.Thr22Ala)	c.95C>T (p.Thr32Ile)	c.97C>T (p.Arg33Trp)	c.226C>T (p.Arg76*)	c.74C>G (p.Ser25*)	c.2000A>G (p.Lys667Arg)
dbSNP	rs77121243	rs16910526	rs193922688	rs28940279	rs28940574	rs116107386	rs149183052	rs78536455	rs138292988	rs202210819	rs121434305	rs200879436
ALL	0.0150 (228/15182)	0.0572 (869/15190)	0.000612 (9/14702)	0.000197 (3/15190)	0.000132 (2/15190)	0.00889 (126/14180)	0.00113 (16/14098)	0.00459 (65/14166)	0.00776 (109/14054)	0.000357 (5/14024)	0.000071 (1/14160)	0.0039 (56/14226)
1 in _	66.6	17.5	1633.6	5063.3	7595	112.5	881.1	217.9	128.9	2804.8	14160	254
NHLBI ALL	0.0138 (179/12998)	0.0611 (795/13006)	0.000719 (9/12518)	0.000231 (3/13006)	0.000154 (2/13006)	0.00959 (115/11996)	0.00109 (13/11914)	0.00484 (58/11982)	0.00859 (102/11870)	0.000422 (5/11840)	0.0000835 (1/11976)	0.00415 (50/12042)
EA	0.000233 (2/8596)	0.0790 (679/8600)	0.00109 (9/8254)	0.000233 (2/8600)	0.000233 (2/8600)	0.0128 (105/8232)	0.00134 (11/8188)	0.00688 (57/8280)	0.0116 (95/8174)	0.000611 (5/8180)	0.000122 (1/8222)	0.000122 (1/8230)
AA	0.0402 (177/4402)	0.0263 (116/4406)	0 (0/4264)	0.000227 (1/4406)	0 (0/4406)	0.00266 (10/3764)	0.000537 (2/3726)	0.000270 (1/3702)	0.00189 (7/3696)	0 (0/3660)	0 (0/3754)	0.0129 (49/3812)
1000G ALL	0.0224 (49/2184)	0.0339 (74/2184)	0 (0/2184)	0 (0/2184)	0 (0/2184)	0.00504 (11/2184)	0.00137 (3/2184)	0.00321 (7/2184)	0.00321 (7/2184)	0 (0/2184)	0 (0/2184)	0.00275 (6/2184)
AFR	0.0915 (45/492)	0.0142 (7/492)	0 (0/492)	0 (0/492)	0 (0/492)	0.00407 (2/492)	0 (0/492)	0 (0/492)	0 (0/492)	0 (0/492)	0 (0/492)	0.0122 (6/492)
AMR	0.0110 (4/362)	0.0442 (16/362)	0 (0/362)	0 (0/362)	0 (0/362)	0.00276 (1/362)	0.00276 (1/362)	0.00552 (2/362)	0.00588 (1/362)	0 (0/362)	0 (0/362)	0 (0/362)
ASN	0 (0/572)	0 (0/572)	0 (0/572)	0 (0/572)	0 (0/572)	0 (0/572)	0 (0/572)	0 (0/572)	0 (0/572)	0 (0/572)	0 (0/572)	0 (0/572)
EUR	0 (0/758)	0.0673 (51/758)	0 (0/758)	0 (0/758)	0 (0/758)	0.0106 (8/758)	0.00264 (2/758)	0.00660 (5/758)	0.00792 (6/758)	0 (0/758)	0 (0/758)	0 (0/758)
ASW	0.0246 (3/122)	0.0328 (4/122)	0 (0/122)	0 (0/122)	0 (0/122)	0.0164 (2/122)	0 (0/122)	0 (0/122)	0 (0/122)	0 (0/122)	0 (0/122)	0 (0/122)
CEU	0 (0/170)	0.0824 (14/170)	0 (0/170)	0 (0/170)	0 (0/170)	0.0118 (2/170)	0 (0/170)	0.0118 (2/170)	0.00588 (1/170)	0 (0/170)	0 (0/170)	0 (0/170)
CHB	0 (0/194)	0 (0/194)	0 (0/194)	0 (0/194)	0 (0/194)	0 (0/194)	0 (0/194)	0 (0/194)	0 (0/194)	0 (0/194)	0 (0/194)	0 (0/194)
CHS	0 (0/200)	0 (0/200)	0 (0/200)	0 (0/200)	0 (0/200)	0 (0/200)	0 (0/200)	0 (0/200)	0 (0/200)	0 (0/200)	0 (0/200)	0 (0/200)
CLM	0.00833 (1/120)	0.0417 (5/120)	0 (0/120)	0 (0/120)	0 (0/120)	0 (0/120)	0 (0/120)	0.0167 (2/120)	0 (0/120)	0 (0/120)	0 (0/120)	0 (0/120)
FIN	0 (0/186)	0.0538 (10/186)	0 (0/186)	0 (0/186)	0 (0/186)	0.00538 (1/186)	0 (0/186)	0 (0/186)	0.00538 (1/186)	0 (0/186)	0 (0/186)	0 (0/186)
GBR	0 (0/178)	0.0787 (14/178)	0 (0/178)	0 (0/178)	0 (0/178)	0.00562 (1/178)	0 (0/178)	0.0112 (2/178)	0.0169 (3/178)	0 (0/178)	0 (0/178)	0 (0/178)
IBS	0 (0/28)	0.0357 (1/28)	0 (0/28)	0 (0/28)	0 (0/28)	0.071 (2/28)	0 (0/28)	0 (0/28)	0.0357 (1/28)	0 (0/28)	0 (0/28)	0 (0/28)
JPT	0 (0/178)	0 (0/178)	0 (0/178)	0 (0/178)	0 (0/178)	0 (0/178)	0 (0/178)	0 (0/178)	0 (0/178)	0 (0/178)	0 (0/178)	0 (0/178)
LWK	0.0979 (19/194)	0.0155 (3/194)	0 (0/194)	0 (0/194)	0 (0/194)	0 (0/194)	0 (0/194)	0 (0/194)	0 (0/194)	0 (0/194)	0 (0/194)	0 (0/194)
MXL	0 (0/132)	0.0606 (8/132)	0 (0/132)	0 (0/132)	0 (0/132)	0 (0/132)	0 (0/132)	0 (0/132)	0 (0/132)	0 (0/132)	0 (0/132)	0 (0/132)
PUR	0.0273 (3/110)	0.0272 (3/110)	0 (0/110)	0 (0/110)	0 (0/110)	0.00909 (1/110)	0.00909 (1/110)	0 (0/110)	0.00909 (1/110)	0 (0/110)	0 (0/110)	0 (0/110)
TSI	0 (0/196)	0.0612 (12/196)	0 (0/196)	0 (0/196)	0 (0/196)	0.0102 (2/196)	0.0102 (2/196)	0.00510 (1/196)	0 (0/196)	0 (0/196)	0 (0/196)	0 (0/196)
YRI	0.131 (23/176)	0 (0/176)	0 (0/176)	0 (0/176)	0 (0/176)	0 (0/176)	0 (0/176)	0 (0/176)	0 (0/176)	0 (0/176)	0 (0/176)	0.0341 (6/176)
		9			10			11	12	13	14	15
Disease name		Pontocerebellar hypoplasia type 1B (PCH1B)			Miller syndrome			Facial dysmorphism, lens dislocation, anterior-segment abnormalities, and spontaneous filtering blebs (FDLAB, or Traboulsi syndrome)	Carpenter syndrome 1 (CRPT1)	Glucocorticoid deficiency 4 (GCCD4)	Aicardi-Goutières syndrome 6 (AGS6)	MEGDEL syndrome
OMIM entry		#614678			#263750			#601552	#201000	#614736	#615010	#614739
Gene name		*EXOSC3*			*DHODH*			*ASPH*	*RAB23*	*NNT*	*ADAR*	*SERAC1*
RefSeqGene		NG_032780.1			NG_016271.1			NG_013210.1	NG_012170.1	NG_032869.1	NG_011844.1	NG_032889.1
mRNA ID		NM_001002269.1			NM_001361.4			NM_004318.3	NM_016277.4	NM_012343.3	NM_001111.4	NM_032861.3
Mutation	c.2034T>G (p.Tyr678*)	c.238G>T (p.Val80Phe)	c.395A>C (p.Asp132Ala)	c.475-1269A>G	c.403C>T (p.Arg135Cys)	c.454G>A (p.Gly152Arg)	c.1036C>T (p.Arg346Trp)	c.2203C>T (p.Arg735Trp)	c.434T>A (p.Leu145*)	c.1990G>A (p.Gly664Arg)	c.577C>A (p.Pro193Ala)	c.1627_1628insTC (p.Ser543Phefs*44)
dbSNP	rs182018947	rs374550999	rs141138948	rs370087266	rs201230446	rs267606766	rs201947120	rs374385878	rs121908171	rs371979800	rs145588689	-
ALL	0.000688 (10/14526)	0.0000660 (1/15154)	0.000724 (11/15190)	0.0000658 (1/15190)	0.000352 (5/14206)	0.0000696 (1/14364)	0.000137 (2/14598)	0.0000658 (1/15190)	0.000395 (6/15190)	0.0000658 (1/15190)	0.00283 (43/15190)	0.0000680 (1/14702)
1 in _	1452.6	15154	1380.9	15190	2841.2	14364	7299	15190	2531.7	15190	353.3	14702
NHLBI ALL	0.000729 (9/12342)	0.0000771 (1/12970)	0.000846 (11/13006)	0.0000769 (1/13006)	0.000416 (5/12022)	0.0000821 (1/12180)	0.000161 (2/12414)	0.0000769 (1/13006)	0.000308 (4/12994)	0.0000769 (1/13006)	0.00315	0.0000799 (1/12518)
EA	0.000239 (2/8366)	0.000116 (1/8586)	0.00128 (11/8600)	0.000116 (1/8600)	0.000607 (5/8232)	0.000120 (1/8302)	0.000239 (2/8362)	0.000116 (1/8600)	0.000233 (2/8590)	0.000116 (1/8600)	0.00372 (32/8600)	0.000121 (1/8254)
AA	0.00176 (7/3976)	0 (0/4384)	0 (0/4406)	0 (0/4406)	0 (0/3790)	0 (0/3878)	0 (0/4052)	0 (0/4406)	0.00454 (2/4404)	0 (0/4406)	0.00204 (9/4406)	0 (0/4264)
1000G ALL	0.000458 (1/2184)	0 (0/2184)	0 (0/2184)	0 (0/2184)	0 (0/2184)	0 (0/2184)	0 (0/2184)	0 (0/2184)	0.000916 (2/2184)	0 (0/2184)	0.000916 (2/2184)	0 (0/2184)
AFR	0.00203 (1/492)	0 (0/492)	0 (0/492)	0 (0/492)	0 (0/492)	0 (0/492)	0 (0/492)	0 (0/492)	0 (0/492)	0 (0/492)	0 (0/492)	0 (0/492)
AMR	0 (0/362)	0 (0/362)	0 (0/362)	0 (0/362)	0 (0/362)	0 (0/362)	0 (0/362)	0 (0/362)	0 (0/362)	0 (0/362)	0.00276 (1/362)	0 (0/362)
ASN	0 (0/572)	0 (0/572)	0 (0/572)	0 (0/572)	0 (0/572)	0 (0/572)	0 (0/572)	0 (0/572)	0 (0/572)	0 (0/572)	0 (0/572)	0 (0/572)
EUR	0 (0/758)	0 (0/758)	0 (0/758)	0 (0/758)	0 (0/758)	0 (0/758)	0 (0/758)	0 (0/758)	0.00264 (2/758)	0 (0/758)	0.00132 (1/758)	0 (0/758)
ASW	0 (0/122)	0 (0/122)	0 (0/122)	0 (0/122)	0 (0/122)	0 (0/122)	0 (0/122)	0 (0/122)	0 (0/122)	0 (0/122)	0 (0/122)	0 (0/122)
CEU	0 (0/170)	0 (0/170)	0 (0/170)	0 (0/170)	0 (0/170)	0 (0/170)	0 (0/170)	0 (0/170)	0.00588 (1/170)	0 (0/170)	0 (0/170)	0 (0/170)
CHB	0 (0/194)	0 (0/194)	0 (0/194)	0 (0/194)	0 (0/194)	0 (0/194)	0 (0/194)	0 (0/194)	0 (0/194)	0 (0/194)	0 (0/194)	0 (0/194)
CHS	0 (0/200)	0 (0/200)	0 (0/200)	0 (0/200)	0 (0/200)	0 (0/200)	0 (0/200)	0 (0/200)	0 (0/200)	0 (0/200)	0 (0/200)	0 (0/200)
CLM	0 (0/120)	0 (0/120)	0 (0/120)	0 (0/120)	0 (0/120)	0 (0/120)	0 (0/120)	0 (0/120)	0 (0/120)	0 (0/120)	0.00833 (1/120)	0 (0/120)
FIN	0 (0/186)	0 (0/186)	0 (0/186)	0 (0/186)	0 (0/186)	0 (0/186)	0 (0/186)	0 (0/186)	0 (0/186)	0 (0/186)	0 (0/186)	0 (0/186)
GBR	0 (0/178)	0 (0/178)	0 (0/178)	0 (0/178)	0 (0/178)	0 (0/178)	0 (0/178)	0 (0/178)	0.00562 (1/178)	0 (0/178)	0 (0/178)	0 (0/178)
IBS	0 (0/28)	0 (0/28)	0 (0/28)	0 (0/28)	0 (0/28)	0 (0/28)	0 (0/28)	0 (0/28)	0 (0/28)	0 (0/28)	0 (0/28)	0 (0/28)
JPT	0 (0/178)	0 (0/178)	0 (0/178)	0 (0/178)	0 (0/178)	0 (0/178)	0 (0/178)	0 (0/178)	0 (0/178)	0 (0/178)	0 (0/178)	0 (0/178)
LWK	0.00515 (1/194)	0 (0/194)	0 (0/194)	0 (0/194)	0 (0/194)	0 (0/194)	0 (0/194)	0 (0/194)	0 (0/194)	0 (0/194)	0 (0/194)	0 (0/194)
MXL	0 (0/132)	0 (0/132)	0 (0/132)	0 (0/132)	0 (0/132)	0 (0/132)	0 (0/132)	0 (0/132)	0 (0/132)	0 (0/132)	0 (0/132)	0 (0/132)
PUR	0 (0/110)	0 (0/110)	0 (0/110)	0 (0/110)	0 (0/110)	0 (0/110)	0 (0/110)	0 (0/110)	0 (0/110)	0 (0/110)	0 (0/110)	0 (0/110)
TSI	0 (0/196)	0 (0/196)	0 (0/196)	0 (0/196)	0 (0/196)	0 (0/196)	0 (0/196)	0 (0/196)	0 (0/196)	0 (0/196)	0.00510 (1/196)	0 (0/196)
YRI	0 (0/176)	0 (0/176)	0 (0/176)	0 (0/176)	0 (0/176)	0 (0/176)	0 (0/176)	0 (0/176)	0 (0/176)	0 (0/176)	0 (0/176)	0 (0/176)

### Disease carrier states of Mendelian disorders

As expected, among 15 genetic diseases detected, the most common was SCA, with a frequency of 1 in 66.6 (1.50%) (Table 1). In contrast, MCPH1 was the rarest disorder, with a frequency of 1 in 14,160 (0.0071%). In addition, carrier prediction unexpectedly revealed high carrier rates (1 in 254.0) for *CEP152* mutations for SCKL5. Carrier statistics are fully reported in Table 1.

### Carrier rate variability by race and ethnicity

**Fig 2:**
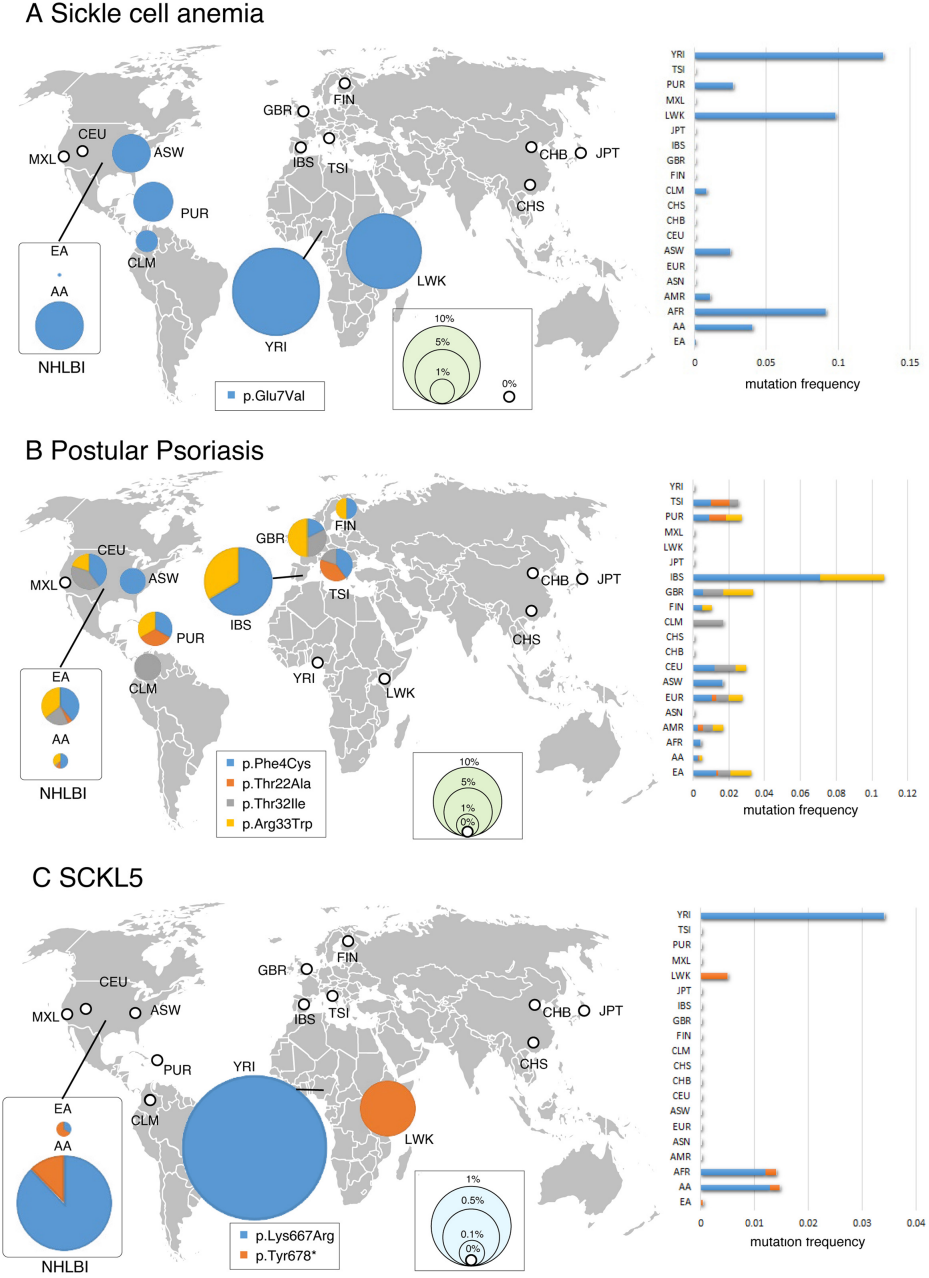
Geographical minor allele frequency distribution for the causative mutations of representative three Mendelian disorders. Pie areas are proportional to the minor allele frequency of the causative mutations for three inherited diseases (A: SCA, B: Pustular psoriasis, C: Miller syndrome). 1000G and NHLBI (2 + 14) populations are displayed separately. The thick white circle indicates the absence (0%) of mutations in the population. The right bar chart shows the mutation minor allele frequency in each population. A world map was obtained from Free Editable Worldmap (http://free-editable-worldmap-for-powerpoint.en.softonic.com/) and modified.

Carrier frequencies for disease-causing mutations varied significantly by racial and ethnic groups although the sample size is not so large in Hispanics and Asians [7, 9, 10]. Fig 2 shows the global map of carrier distribution of eight causative mutations for three Mendelian disorders. For example, an average of 0.11% of individuals were carriers for Miller syndrome, but the frequency ranged from 0.18% of European individuals to 0% of Africans, Asians, and Hispanics (Fig 2C). For ethnic groups such as European, this higher frequency was unreported before and thus suggest that the European population is right target for screening for Miller syndrome. Among 15,190 haploid exomes, causative alleles for seven disorders (SCA, SCKL5, Primary immunodeficiency, Canavan disease, Pustular psoriasis, CRPT1 and AGS6) were more or less prevalent in both African and European populations (Fig 2 and Table 1). In contrast, mutations for the other eight disorders (CPHD2, RCD, MCPH1, PCH1B, Miller syndrome, FDLAB, GCCD4 and MEGDEL syndrome) were observed only in Europeans while they were not detected in other populations. There were no carriers for any of the 18 inherited disorders among the dataset from Asian populations.

### Estimated carrier rates correspond to those seen in clinical practice

SCA is an inherited blood-related disorder that affects hemoglobin and is characterized primarily by chronic anemia and periodic pain episodes [18, 19]. A mutation in the *HBB* gene, commonly called Hemoglobin S (HbS), causes SCA [18, 19]. SCA is common among persons whose ancestors descended from tropical regions, particularly Sub-Saharan Africa, South America, Saudi Arabia, India, and Mediterranean countries (e.g. Italy, Greece, and Turkey) [18, 19]. The CDC has reported that in the United States, SCA affects approximately 90,000–100,000 persons, most of whom have ancestors of African descent [20]. The disease occurs in about 1 in every 500 African-American births and 1 in every 36,000 [20] (or 1,000–1,400 [21]; the incidence rate is controversial) Hispanic-American births. However, highly accurate epidemiological studies based on clinical practice are still rare.

Table 2 shows, for 14 + 2 ethnic groups in the dataset, my estimates and literature estimates for carrier frequency for SCA. Predicted carrier rates were not statistically different from clinical geographic prevalence [20–22] and Bayesian geostatistical map of HbS allele [23] (Table 2). There were no notable outliers, but I observed significantly higher carrier frequencies than expected for SCA in two populations (LWK (1000G, 9.79%) and PUR (1000G, 2.73%)) (*P* < 0.01) (Table 2). It is possible that the collected population was geographically distinct at these loci relative to prior studies. As expected, the HbS allele in African populations (NHLBI, 4.02%; 1000G, 9.15%) was detected at a significantly higher rate than in all European populations (NHLBI, 0.0233%; 1000G, 0%), Hispanics (1000G, 1.10%), and Asians (1000G, 0%) (*P* < 0.01) (Table 2).

**Table 2:** Comparison of predicted exome-based carrier rates with previous clinical estimates. The *P*-value is calculated from Chi-square tests between two carrier estimates.

			Allele frequency		
	Ethnic group	Country	This study	Previous study (+reference)	P value
NHLBI	EA	USA	0.000233 (2/8596)	-	-
	AA	USA	0.0402 (177/4402)	0.0447–0.0577 (*1)	6.46E-07-0.149
1000G	AFR		0.0915 (45/492)	0.0447–0.0577 (*1)	12.7E-05-0.0013
	AMR		0.0110 (4/362)	0.00527–0.0316 (*1,2)	0.0206–0.252
	ASN		0 (0/572)	0 (*3,4)	NA
	EUR		0 (0/758)	-	-
	ASW	USA	0.0246 (3/122)	0.0447–0.0577 (*1)	0.148–0.401
	CEU	USA	0 (0/170)	-	-
	CHB	China	0 (0/194)	0 (*4)	NA
	CHS	China	0 (0/200)	0 (*4)	NA
	CLM	Colombia	0.00833 (1/120)	0.008 (*4)	0.967
	FIN	Finland	0 (0/186)	0 (*4)	NA
	GBR	England	0 (0/178)	0.009 (*4)	0.204
	IBS	Spain	0 (0/28)	0.007 (*4)	0.657
	JPT	Japan	0 (0/178)	0 (*4)	NA
	LWK	Kenya	0.0979 (19/194)	0.038 (*4)	0.000346
	MXL	Mexico	0 (0/132)	0.007 (*4)	0.335
	PUR	Puerto Rico	0.0273 (3/110)	0.004 (*4)	0.0001
	TSI	Italy	0 (0/196)	0.005 (*4)	0.321
	YRI	Nigeria	0.131 (23/176)	0.171 (*4)	0.155

Previously reported carrier rates are derived from four references (*1; National Center for Disease Control (http://www.cdc.gov/ncbddd/sicklecell/data.html), *2; Morton DA. *Medical Issues in Social Security Disability* 2013; *3, Modell B, et al. *Bull World Health Organ* 2008; *4, Piel FB, et al. *Lancet* 2013).

The prevalence rates of SCA in Hispanic Americans are controversial (1 in 36,000 [20] or 1 in 1,000–1,400 [21]), but the projected carrier rate here could support both data depending on the ancestral origin (Table 2). Taken together, exome-based estimates corresponded to those in the clinical prevalence survey and represented equivalent accuracy that may be achievable in clinical practice.

### Screening priority for genetic testing

Current genetic testing is generally performed according to the ranking of carrier rates of the target mutations. Yet, precise data of targeted panel of genetic testing is not sufficient in clinical practice due to the large number of rare disorders. This tendency is particularly true for recently identified causative genes. Here, I demonstrated that the exome-based methods made it possible to identify a small number of high-priority nonsense and missense mutations linked to genetic disorders (Table 1). For example, the data suggests that, among six causative mutations for PCH1B, only one mutation (p.Asp132Ala,) should be high priority for *EXOSC3* mutation screening in European populations, whereas other mutations are speculated to be quite rare (Table 1). The ranking of carrier rates of mutations was as follows: p.Asp132Ala (NHLBI EA, 0.128%) > p.Val80Phe (0.0116%) = c.475-1269A>G (0.0116%) > other mutations (0%). In the case of Miller syndrome, for which mutations have been reported in several papers, three mutations [p.Arg135Cys (NHLBI EA, 0.0607%), p.Gly152Arg (0.120%), and p.Arg346Try (0.161%)] should be given first priority for *DHODH* mutation screening in Europeans but not Africans. A different tendency was obtained for SCKL5: two mutations [p.Lys667Arg (NHLBI AA, 1.29%) and p.Tyr678* (0.176%)] occupied a central position in African populations (Table 1). These frequent mutations were detected in the 1000G dataset [p.Lys667Arg (1000G AFR, 1.22%) and p.Tyr678* (0.203%)]. Taken together, these data will allow the formulation of a suitable mutation panel that can be applied to determine the priority of genetic testing in clinical practice.

I further searched for undetected mutations using the Exome Aggregation Consortium (ExAC), which summarizes and categorizes exome data of 60,706 unrelated individuals from a variety of large-scale sequencing projects into six races (Table 3). The ExAC dataset detected additional 29 mutations although this data did not provide country-by-country genetic epidemiology of inherited disorders (Table 3). This result suggested that larger sample sizes and/or combinational use of a set of large exome sequencing projects could allow for more accurate prediction of carrier rates.

**Table 3:** Estimated carrier rates of 17 Mendelian disorders using ExAC data. The carrier rates of Mendelian disorders were estimated using ExAC dataset. Child-hood cardiomyopathy (MIM no description) and Usher syndrome type 1J (USH1J) (#614869) were detected in ExAC but not in 1000G and NHLBI. ExAC populations are largely divided into six races: African, Latino, European (non-Finnish), European (Finnish), South Asian, East Asian, and Other.

	1	2	3						4					5
Disease name	SCA	Primary immunodeficiency	CPHD2						Canavan disease					Pustular psoriasis
OMIM entry	#603903	#613108	#262600						#271900					No description
Gene name	*HBB*	*CLEC7A*	*PROP1*						*ASPA*					*AP1S3*
Mutation	c.20A>T (p.Glu7Val)	c.714T>G (p.Tyr238*)	c.217C>T (p.Arg73Cys)	c.218G>A (p.Arg73His)	c.296G>A (p.Arg99Gln)	c.301_302delAG (p.Leu102Cysfs*8)	c.349T>A (p.Phe117Ile)	c.358C>T (p.Arg120Cys)	c.212G>A (p.Arg71His)	c.746A>T (p.Asp249Val)	c.854A>C (p.Glu285Ala)	c.876delAGAA (p.Glu293Leufs*8)	c.914C>A (p.Ala305Glu)	c.11T>G (p.Phe4Cys)
dbSNP	rs77121243	rs16910526	rs121917843	rs121917842	rs137853100	rs193922688	rs121917840	rs121917839	rs104894553	rs104894552	rs28940279		rs28940574	rs116107386
African	0.0485 (505/10404)	0.0243 (253/10404)	0 (0/10184)	0 (0/10184)	0 (0/10006)	0 (0/9980)	0 (0/8952)	0.000203 (0/9864)	0.000203 (0/10402)	0 (0/7694)	0.0000967 (1/10344)	0 (0/10350)	0.000301 (3/9962)	0.00350 (34/9718)
Latino	0.00104 (12/11548)	0.0341 (395/11578)	0 (0/11548)	0 (0/11548)	0.0000866 (1/11554)	0 (0/11548)	0 (0/9124)	0 (0/11548)	0 (0/11574)	0 (0/6726)	0 (0/11514)	0 (0/11512)	0.0000891 (1/11218)	0.00480 (55/11450)
South Asian	0.000545 (9/16512)	0.00965 (1593/16510)	0 (0/16510)	0 (0/16510)	0 (0/16506)	0 (0/16504)	0 (0/14108)	0 (0/14586)	0 (0/16510)	0 (0/9492)	0 (0/16344)	0 (0/16342)	0 (0/15818)	0.00195 (30/15394)
European (Non-Finnish)	0.0000899 (6/66734)	0.0000899 (6/66734)	0.0000152 (1/65894)	0.0000304 (2/65864)	0.0000304 (2/65844)	0.000259 (17/65770)	0.000266 (15/56442)	0.0000675 (4/59246)	0.000150 (10/66732)	0.000336 (14/41652)	0.000633 (42/66354)	0.0000151 (1/66340)	0.000358 (23/64266)	0.0118 (778/65728)
East Asian	0 (0/8620)	0 (0/8620)	0 (0/8612)	0 (0/8610)	0.000116 (1/8612)	0 (0/8602)	0 (0/7392)	0 (0/7802)	0 (0/8642)	0 (0/5214)	0 (0/8620)	0 (0/8612)	0 (0/8424)	0.000117 (1/8580)
European (Finnish)	0 (0/6614)	0 (0/6614)	0 (0/6590)	0 (0/6592)	0 (0/6608)	0 (0/6608)	0 (0/4968)	0 (0/5272)	0.00197 (13/6614)	0 (0/4638)	0 (0/6606)	0 (0/6604)	0 (0/6502)	0.00319 (21/6574)
Other	0 (0/908)	0 (0/908)	0 (0/886)	0 (0/886)	0 (0/900)	0 (0/898)	0 (0/740)	0 (0/776)	0 (0/908)	0.00159 (1/628)	0 (0/900)	0 (0/898)	0 (0/876)	0.00801 (7/874)
					6	7			8		9			
Disease name					RCD	MCPH1			SCKL5		PCH1B			
OMIM entry					#615780	#251200			#613823		#614678			
Gene name					*PLK1S1*	*MCPH1*			*CEP152*		*EXOSC3*			
Mutation	c.64A>G (p.Thr22Ala)	c.95C>T (p.Thr32Ile)	c.97C>T (p.Arg33Trp)	c.248T>C (p.Ile83Thr)	c.226C>T (p.Arg76*)	c.74C>G (p.Ser25*)	c.215C>T (p.Ser72Leu)	c.305C>G (p.Ser101*)	c.2000A>G (p.Lys667Arg)	c.2034T>G (p.Tyr678*)	c.2T>C (p.Met1?)	92G>C (p.Gly31Ala)	c.238G>T (p.Val80Phe)	c.395A>C (p.Asp132Ala)
dbSNP	rs149183052	rs78536455	rs138292988	rs202157374	rs202210819	rs121434305	rs387906961		rs200879436	rs182018947		rs387907196	rs374550999	rs141138948
African	0.000204 (2/9800)	0.000714 (7/9800)	0.00133 (13/9798)	0.000103 (1/9704)	0 (0/4932)	0 (0/10184)	0 (0/9802)	0 (0/9608)	0.0136 (132/9692)	0.00173 (17/9806)	0 (0/6472)	0 (0/8272)	0 (0/6932)	0.0000961 (1/10404)
Latino	0.000867 (10/11534)	0.00390 (45/11536)	0.00625 (72/11526)	0 (0/11404)	0.00616 (12/1948)	0 (0/11548)	0.0000865 (1/11564)	0.0000869 (1/11504)	0.000174 (2/11466)	0.0000864 (1/11576)	0 (0/8778)	0 (0/10236)	0 (0/8806)	0.000259 (3/11576)
South Asian	0.000606 (10/16498)	0.000545 (1/16486)	0.00455 (75/16482)	0.0124 (201/16214)	0 (0/9454)	0 (0/16510)	0 (0/16510)	0 (0/16438)	0.0000610 (1/16404)	0.000545 (9/16508)	0.000503 (6/11938)	0 (0/14680)	0.000499 (7/14042)	0.0000606 (1/16510)
European (Non-Finnish)	0.00167 (111/66662)	0.00527 (351/66650)	0.0109 (724/66632)	0 (0/65634)	0.00134 (30/22330)	0.0000304 (2/65864)	0.0000150 (1/66736)	0 (0/66164)	0.0000151 (1/66196)	0.000150 (10/65864)	0 (0/483464)	0 (0/57660)	0.0000401 (2/49814)	0.000510 (34/66726)
East Asian	0 (0/8614)	0 (0/8610)	0 (0/8604)	0 (0/8582)	0 (0/3338)	0 (0/8610)	0 (0/8626)	0 (0/8586)	0 (0/8586)	0.000348 (3/8624)	0 (0/6844)	0 (0/7842)	0 (0/6616)	0 (0/8654)
European (Finnish)	0 (0/6610)	0.00166 (11/6612)	0.0101 (67/6608)	0 (0/6554)	0 (0/2814)	0 (0/6592)	0 (0/6612)	0 (0/6614)	0 (0/6600)	0 (0/6614)	0 (0/3528)	0 (0/4366)	0 (0/3730)	0 (0/6614)
Other	0.00111 (1/900)	0.00111 (1/900)	0.0122 (11/900)	0 (0/888)	0.00267 (1/374)	0 (0/896)	0 (0/900)	0 (0/888)	0.00112 (1/890)	0 (0/900)	0 (0/602)	0.00142 (1/704)	0 (0/616)	0 (0/908)
		10								11	12		13	
Disease name		Miller syndrome								FDLAB	CRPT1		GCCD4	
OMIM entry		#263750								#601552	#201000		#614736	
Gene name		*DHODH*								*ASPH*	*RAB23*		*NNT*	
Mutation	c.475-1269A>G	c.56G>C (p.Gly19Glu)	c.403C>T (p.Arg135Cys)	c.454G>A (p.Gly152Arg)	c.595C>T (p.Arg199Cys)	c.605G>C (p.Gly202Asp)	c.976C>T (p.Arg326*)	c.1036C>T (p.Arg346Trp)	1175A>G (p.Asp392Gly)	c.2203C>T (p.Arg735Trp)	c.83G>A (p.Arg28*)	c.434T>A (p.Leu145*)	c.1990G>A (p.Gly664Arg)	c.3027T>G (p.Asn1009Lys)
dbSNP	rs370087266	rs267606765	rs201230446	rs267606766	rs267606769	rs267606767		rs201947120		rs374385878	rs376394715	rs121908171	rs371979800	rs370273690
African	0 (0/10106)	0 (0/9800)	0 (0/9744)	0 (0/9800)	0 (0/6920)	0 (0/7102)	0 (0/9624)	0 (0/9780)	0 (0/9808)	0.0000970 (1/10310)	0.0000961 (1/10402)	0.000194 (2/10318)	0 (0/10396)	0 (0/11322)
Latino	0 (0/11494)	0.000518 (6/11576)	0.000261 (3/11516)	0 (0/11574)	0 (0/7566)	0 (0/8048)	0 (0/11576)	0.0000432 (5/11574)	0 (0/11570)	0 (0/11412)	0 (0/11570)	0.000177 (2/11328)	0 (0/11558)	0 (0/11558)
South Asian	0 (0/16178)	0 (0/16512)	0.0000606 (1/16494)	0 (0/16512)	0 (0/12628)	0.000232 (3/12926)	0 (0/16512)	0 (0/16512)	0 (0/16512)	0 (0/16216)	0 (0/16510)	0 (0/16122)	0 (0/16496)	0 (0/16280)
European (Non-Finnish)	0.0000303 (2/65970)	0 (0/66732)	0.000631 (42/66518)	0.000180 (12/66736)	0.0000621 (3/48338)	0.0000397 (2/50334)	0.0000301 (2/66458)	0 (0/66732)	0.0000450 (3/66722)	0.0000453 (3/66256)	0.0000300 (2/66724)	0.000447 (29/64878)	0.0000150 (1/66702)	0.0000152 (1/65808)
East Asian	0 (0/8610)	0 (0/8626)	0 (0/8612)	0 (0/8626)	0 (0/6284)	0 (0/6608)	0 (0/8624)	0 (0/8626)	0 (0/8626)	0 (0/8500)	0 (0/8654)	0 (0/8532)	0.000116 (1/8654)	0 (0/8594)
European (Finnish)	0 (0/6602)	0 (0/6614)	0 (0/6590)	0 (0/6614)	0 (0/3280)	0 (0/3488)	0 (0/6614)	0 (0/6614)	0 (0/6610)	0 (0/6552)	0 (0/6614)	0.000152 (1/6560)	0 (0/6608)	0 (0/6568)
Other	0 (0/888)	0 (0/900)	0 (0/894)	0 (0/898)	0 (0/616)	0 (0/634)	0 (0/900)	0 (0/900)	0 (0/902)	0 (0/898)	0 (0/908)	0 (0/888)	0 (0/906)	0 (0/896)
	14		15					16			17			
Disease name	AGS6		MEGDEL syndrome					Childhood-onset dilated cardiomyopathy			USH1J			
OMIM entry	#615010		#614739					no description			#614869			
Gene name	*ADAR*		*SERAC1*					*RAF1*			*CIB2*			
Mutation	c.577C>A (p.Pro193Ala)	c.2615T>C (p.Ile872Thr)	c.202C>T (p.Arg68*)	c.442C>T (p.Arg148*)	c.579delA (p.Leu193Profs*9)	c.1171delGACT (p.Gln390Profs*29)	c.1493G>C (p.Ser498Thr)	c.709G>A (p.Ala237Thr)	c.928A>G (p.Thr310Ala)	c.1923C>T (p.Thr641Met)	c.272T>C (p.Phe91Ser)			
dbSNP	rs145588689	rs398122897	rs529232938	rs387907236			rs201941476				rs397515411			
African	0 (0/10396)	0 (0/10400)	0 (0/10364)	0 (0/10394)	0 (0/10370)	0.000134 (1/7478)	0 (0/10360)	0 (0/10364)	0 (0/10394)	0 (0/10406)	0 (0/10404)			
Latino	0 (0/11578)	0 (0/11564)	0 (0/11568)	0 (0/11578)	0 (0/11560)	0 (0/6722)	0 (0/11560)	0 (0/11346)	0.0000864 (1/11570)	0 (0/11578)	0 (0/11574)			
South Asian	0 (0/16512)	0 (0/16510)	0.0000606 (1/16508)	0 (0/16510)	0 (0/16478)	0 (0/12818)	0 (0/16486)	0 (0/16334)	0 (0/16512)	0 (0/16512)	0.000364 (6/16508)			
European (Non-Finnish)	0.0000150 (1/66740)	0.0000150 (1/66718)	0.0000150 (1/66666)	0.0000150 (1/66722)	0.0000300 (2/66674)	0 (0/43262)	0.0000901 (6/66608)	0.0000302 (2/66268)	0.0000150 (1/66624)	0.0000150 (1/66738)	0 (0/66726)			
East Asian	0 (0/8654)	0 (0/8644)	0 (0/8648)	0.000231 (2/8652)	0 (0/8648)	0 (0/5592)	0 (0/8638)	0 (0/8568)	0 (0/8654)	0 (0/8654)	0 (0/8654)			
European (Finnish)	0 (0/6614)	0 (0/6600)	0 (0/6614)	0 (0/6614)	0 (0/6614)	0 (0/4148)	0 (0/6612)	0 (0/6544)	0 (0/6612)	0 (0/6614)	0 (0/6614)			
Other	0 (0/908)	0 (0/906)	0 (0/906)	0 (0/908)	0 (0/908)	0 (0/638)	0 (0/908)	0 (0/898)	0 (0/908)	0 (0/908)	0 (0/906)			

### Consistency of data between two different exome sequencing projects

I next examined the extent of differences in two exome-based carrier rates by comparing carrier rates in African and European ancestries between 1000G and NHLBI datasets. A pair-wise proportions test [24] was used, which was appropriate to test the null hypothesis stating that proportions in the two estimates were significantly different. This formula is referred to as a z-test because the statistic was as follows:

Z=(p1^−p2^)/[p^(1−p^)(1/n1+1/n2)]1/2 (2)

where pˆ = (p_1_ + p_2_)/(n_1_ + n_2_) and the indices (1, 2) refer to the first and second column of the table. A pair-wise proportion test between two exome resources showed no significant differences between the two different exome studies (46 cases; *P* >> 0.05), except in two African cases (*P* < 0.05) (S3 Table). This finding raises the possibility that exome-based predictions are divorced from sources of various arbitrary errors (e.g., diagnostic capacity) and may be an objective indicator.

### Risk simulation and mutation detection rate of autosomal recessive disease

**Fig 3:**
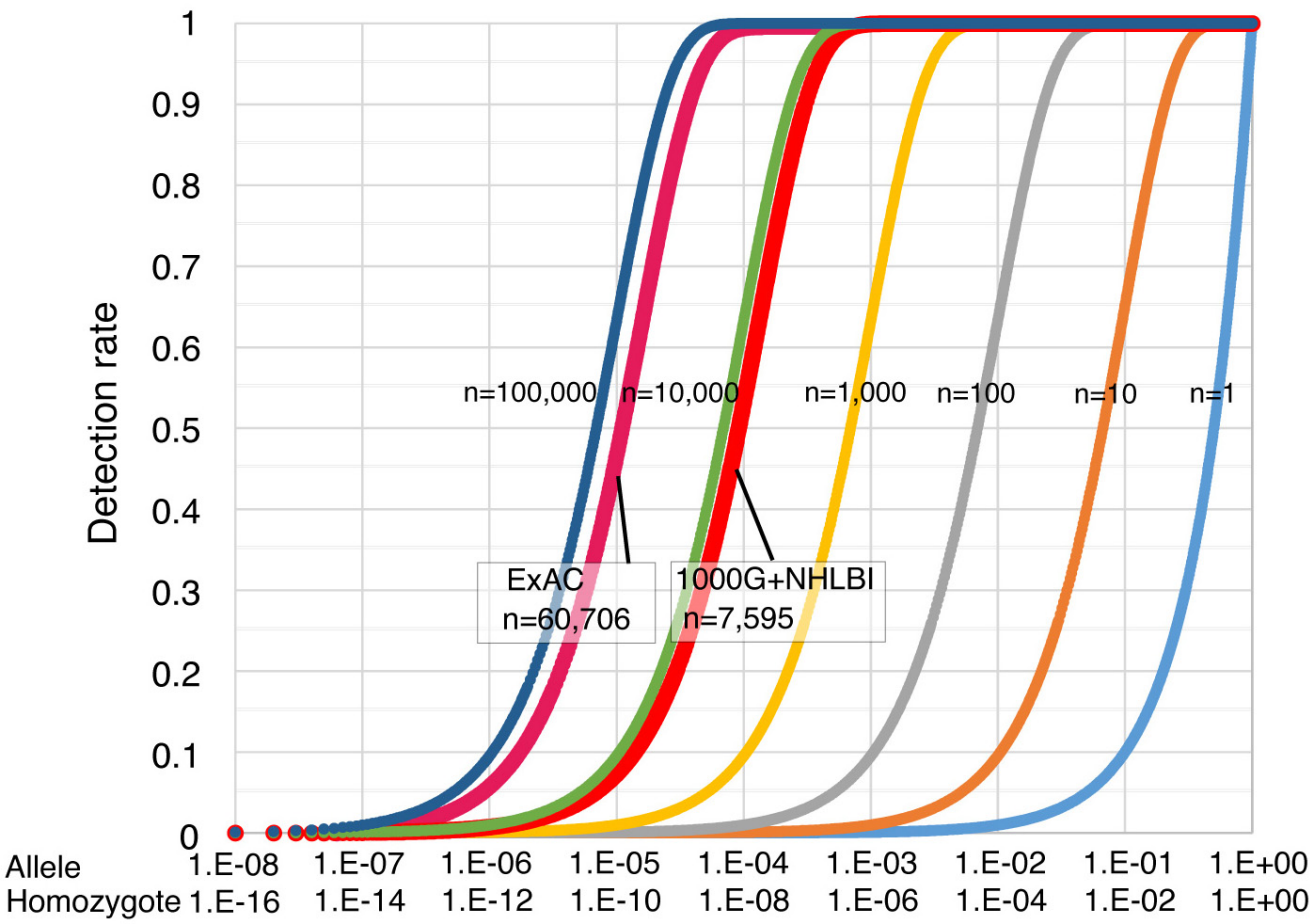
Risk simulation and mutation detection rate of autosomal recessive disease. The simulation graph depicts the theoretical mutation detection probability of high-penetrance genetic mutations (under the condition of *σ* = 0.01) that are associated with inherited disorders. The simulation sample sizes range from 1 to 100,000. The y-axis corresponds to the detection rate of causative mutations.

Finally simple deterministic formulae were introduced to predict the mutation detection rate of genetic risk using exome studies assuming a single-gene disease with an autosomal recessive inheritance pattern. The formula of the mutation detection rate (*D*) of Mendelian disorders was as follows:

$$D=\left[1-{\left\{1-p\left(1-\sigma \right)\right\}}^{N}\right]$$ (3)

where *p* refers to the mutation carrier rate in each population, and σ indicates the error rate of exome sequencing. *N* refers to the number of exomes available for epidemiological analysis. Fig 3 shows the simulation curve for the mutation detection rate. This prediction equation is applicable to general cases of predicting the incidence of inherited disorders. This predictive equation is responsive to parameters that affect carrier rate and data accuracy, and it is independent of the distribution of fitness effects. The epidemiological study was performed using a total of 7,595 samples from NHLBI and 1000G datasets, and a target mutation with carrier rate of 0.001 in this group could be theoretically detected with a probability of 99.95% under the condition of *σ* = 0.01. When the ExAC dataset was used under the same conditions, the probability of undetected rates was 7.70E-25%. Exome sequencing errors now are generally small (*σ* < 0.01) and thus have a small effect on mutation detection rates (S1 Fig).

## Discussion

During the past several decades, biomedical research has identified the causative genes for almost >3,000 Mendelian disorders [1–4]. NGS results have provided empirical evidence that the genetic architecture of Mendelian disease is one of many rare causal mutations, although NGS have not yet identified all genetic mutations [2–4]. Despite the accumulation of significant genetic data, the epidemiology of Mendelian disorders remains unknown. The initial study here demonstrated the structured concept that genetic risk prediction using exome sequences accurately revealed carrier frequencies for rare Mendelian mutations with a small margin of error (Fig 2 and Table 2). The estimation algorithm was successfully applied to developing countries, and showed strong regional specificity of causative alleles (Fig 2). This study also set priorities aligning causative mutations with their carrier rates (Table 2). The accumulation of these data will make it possible to perform closely focused diagnostic genetic tests in specific countries and cities and to plan clinical services, assess priorities, and monitor prevalence trends. I have recently showed that exome-based epidemiology also could have the potential to provide a clue to understand the penetrance of each mutation [25].

A recent exome-based study [26], which focused on common diseases of interest, also successfully performed the risk prediction of target genetic disorders of newborn-screening, age-related macular degeneration (ARMD) and drug response across the two populations (American African and European). Their and my results suggested that NGS data could yield the useful information for applying genetic screening of genetic disorders in clinical practice.

Except Asian populations, the other populations have wider range of genetic variations, and the regional specificity is largest in African populations [14, 15, 27]. Therefore recent analysis [26] about a per-region breakdown of African allele frequency estimates possibly does not reflect the complex genetic structures in African populations. It is rational to analyze country-by-country and ethnicity-by-ethnicity epidemiology by using 1000G (Fig 2).

### Data quality and limitations

The simulation studies here suggest that larger sample sizes or combination studies will allow for more accurate prediction of genetic risk (Fig 3). The ExAC data highlighted usefulness of large population size. Yet note that the present ExAC data also contains individuals sequenced as part of various disease-specific studies and does not reflect the complex genetic structures in African populations.

There were also some logistical issues that must be addressed when performing genetic epidemiological studies. The first limiting factor is consanguineous marriage [28–30], which is irregular from the standpoint of population genetics. This practice largely influences the prevalence rate for autosomal recessive disorders [28, 29]. Most recent studies have used whole-exome sequencing of individuals from consanguineous families to identify rare coding variations in the rare pathogenesis [2–4], and some rare heritable disorders may never occur with outbreeding. Rates of consanguinity (e.g., marriage between cousins) vary greatly between and within countries and regions, but the prevalence is highest in North Africa, the Middle East, and South Asia and among migrant communities in North America, Europe, and Australia [29, 30]. At present, about 20% of the world’s population lives in communities with a preference for consanguineous marriages [29]. Public understanding regarding the genetic risk of consanguinity is still low in these countries [29, 30]http://www.nature.com/ejhg/journal/v22/n4/full/ejhg2013167a.html-bib8 The current accepted belief is that the consanguinity infrequently cause genetic disease, so it is important to provide evidence-based recommendations for genetic counseling and screening for consanguineous couples and not to provoke unnecessary alarm. The research here may promote the diffusion of overview on reproductive risks associated with consanguinity when the sample size are further extended. Intriguingly recent research also provides a fascinating view that the genomic inbreeding coefficient of each individual is an unexpected high to varying degrees even in 1000G data [31].

The second limiting factor is prenatal genetic counseling and testing. SCA, for which the U.S. Preventive Services Task Force (USPSTF) recommends screening [30], is a good example. Recent advances in prenatal genetic diagnosis make it easier than ever to gather more information on individuals prior to their birth [32, 33]. It is, therefore, crucial to consider the potential effect of abortion on the prevalence rates.

The third limiting factor is the mode of inheritance. The initial dataset in this study was originally derived from individuals with no cognitive impairment. Predicting risk has been successful for diseases that follow a simple mode of recessive inheritance, but risk prediction is challenging for autosomal dominant traits in this dataset. To analyze the autosomal dominant disorders, it is necessary to collect general population in specific area independent of their phenotypes.

The fourth limiting factors are the experimental limitations and uncertainties in identifying causative disease mutations. There is often the case where the causative disease-causing mutations are determined too easily without analyzing potential effect of mutations [34, 35] and the population exomes may not have read coverage over all of the causative loci. Some causative mutations may have been previously unreported and would occur *de novo* in the future as the past has already shown [36–39]. In addition the degree of penetrance of the mutations remain largely unknown, and some reported disease mutations may be in fact not disease causing [40]. Therefore the carrier rates could be underestimated or overestimated. I suppose that discordance between carrier and prevalence rates of each mutation could provide a clue to understand the penetrance as well as screening priority.

### Carrier rate in developing countries

One of the greatest merits of exome-based epidemiology is that we can easily conduct a part of public health surveillance of genetic disorders even in developing countries. According to the World Health Organization (WHO), congenital and inherited disorders increasingly contribute to perinatal morbidity and mortality in developing countries [41]. Despite this fact, many countries in Africa, South Asia, and South America still lack national policies and recommendations regarding screening for developmental abnormalities [12]. Genetic epidemiological studies have the potential to provide scientific evidence of genetic risks in most countries and disseminate public health advice. Given the lack of sampling depth in these countries, it seems that the ethnic groups who need the information and counseling the most, have the least sampling. The geographical portfolio of exome-based prediction could be expanded to more disorders and more countries. Furthermore, on this basis, key infrastructure requirements must be placed in sociopolitical frameworks, and medical resources must be allocated for institutions in both developed and developing countries.

## Methods

### Analysis of genetic mutations using two representative population exome projects

Genotyping pipelines from 1000G (Phase 1) (http://www.ncbi.nlm.nih.gov/variation/tools/1000genomes/) and NHLBI (http://www.nhlbi.nih.gov/) projects were collected in VCF format. The dataset consisted of a total of 15,190 haploid exomes from high-coverage exome sequence data derived from 14 + 2 ethnic groups. NHLBI data contains individuals sequenced as part of various disease-specific studies and may not partially reflect the precise genetic population structures while 1000G collected healthy individuals. The validity of a part of the NHLBI dataset was previously assessed by NHLBI using Sanger sequencing [novel singleton variants, 143/145 (99%); novel nonsingleton variants 316/323 (98%)] [17]. The genotype accuracy of 1000G was estimated at 97.4% (20,687/21,235) by comparing with the HapMap genotype calls [15]. The 1000G and NHLBI datasets (VCF files) were filtered on Variant Tools (http://varianttools.sourceforge.net/Annotation/HomePage) and Microsoft Excel by total read depth, the number of individuals with coverage at the site, the fraction of mutation reads in each heterozygote, and the average position of mutation alleles along a read. Eighteen recessively inherited diseases were probatively retrieved and selected from literature (published from 1957 to 2014) and derived from NCBI OMIM (http://www.ncbi.nlm.nih.gov/omim) and PubMed (http://www.ncbi.nlm.nih.gov/pubmed). Causative mutations for inherited disorders were derived from these datasets based on the corresponding chromosome position (UTR, coding, intron, and splice site). ClinVar and HGMD were supplementarily reviewed to collect the mutations. Identified mutations were then classified by mutation type, allele frequency, racial groups, and clinical impact. Information on mutation types, positions, reference sequences, and pathogenicity were retrieved from NCBI dbSNP (http://www.nlm.nih.gov/SNP/) and UCSC genome browser (http://genome.ucsc.edu/) to generate exome-based epidemiology. Statistical analysis, including carrier rate (%), was performed with Excel. ExAC Browser (http://exac.broadinstitute.org/) was additionally searched for the mutation alleles of 18 inherited disorders. A global map of carrier rate distribution was manually constructed for 15 recessive disorders collated from literature sources. A world map was obtained from Free Editable Worldmap (http://free-editable-worldmap-for-powerpoint.en.softonic.com/) and modified.

### Pair-wise proportion tests of data consistency between two different exome resources

To project the performance of risk prediction based on analyses of exome sequence studies, I statistically compared exome-based estimates with the clinical prevalence survey. Evidence of data consistency was based on significant differences in pair-wise comparisons between populations if two estimates differed significantly (two-sample test for equality of proportions with continuity correction). The standard hypothesis test was *H*_*0*_: π_1_ = π_2_ against the alternative (two-sided) *H*_*1*_: π_1_ ≠ π_2_ The pair-wise prop test can be used to test the null hypothesis that the proportions (probabilities of success) in two groups are the same. In a two-way contingency table where *H*_*0*_: π_1_ = π_2_, this should yield comparable results to those of the ordinary χ^2^ test.

### Mutation detection simulation of inherited diseases

To perform mutation detection simulation based on population exome sequences, a deterministic formulae ($D=\left[1-{\left\{1-p\left(1-\sigma \right)\right\}}^{N}\right]$) was calculated to predict the mutation detection rate of genetic risk using exome studies assuming a single-gene disease with an autosomal recessive inheritance pattern. The variable *p* refers to the mutation carrier rate in each population, and *σ* indicates the error rate of exome sequencing. *N* refers to the number of exomes available for epidemiological analysis. The simulation curve for the mutation detection rate is calculated and drawn using the R 3.13 statistical software (http://www.r-project.org/) together with the RColorBrewer package (http://cran.r-project.org/web/packages/RColorBrewer/index.html).

## Supporting Information

S1 Fig Risk simulation and mutation detection rate of autosomal recessive disease. The theoretical mutation detection probability of high-penetrance genetic variants is calculated under the three condition (σ = 0; 0.01; 0.1) although the simulation under σ = 0.1 is unlikely situation. The simulation sample sizes range from 1 to 100,000. The y-axis corresponds to the detection rate of causative mutations.
(PDF)

S1 Table Population disposition (ethnicity and male/female ratio). (PDF)

S2 Table Lists of genetic disorders and their causative genes in this study. Target causative mutation lists analyzed in this study and representative reference lists.
(PDF)

S3 Table Comparison of carrier rates between two different exomes (1000 Genomes and NHLBI). The P-value is calculated from pair-wise proportion tests of allele frequencies in European and African ancestries between the two different exome resources (1000 Genomes vs NHLBI).
(PDF)
