# Supplementary figures and images for: Global Carrier Rates of Rare Inherited Disorders Using Population Exome Sequences
Source: PLoS One. 2016 May 24;11(5):e0155552. doi: 10.1371/journal.pone.0155552 (PMC4878778; doi:10.1371/journal.pone.0155552)

# Figure S1

(a)  $\sigma=0$

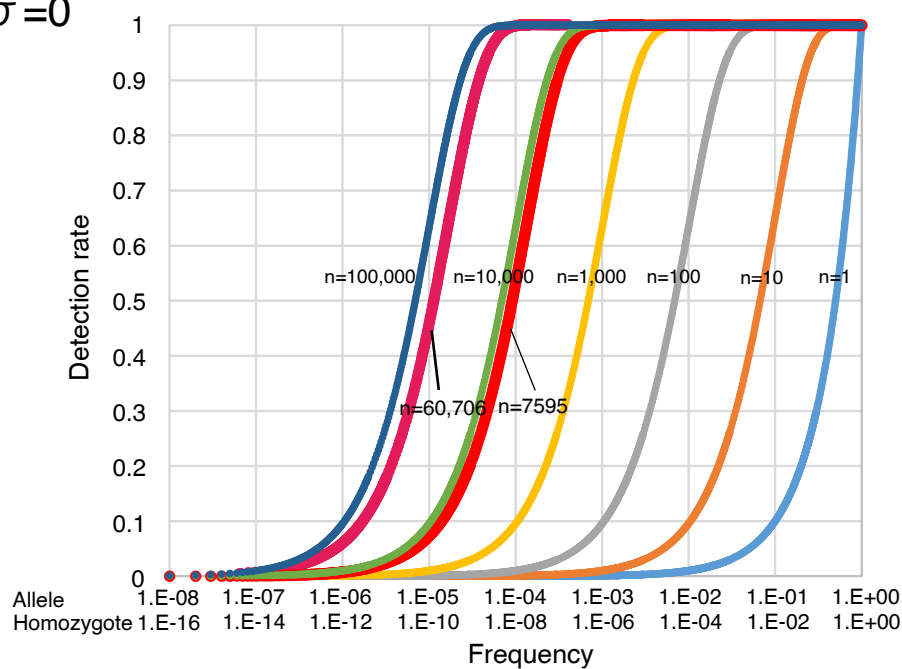

(b)  $\sigma=0.01$

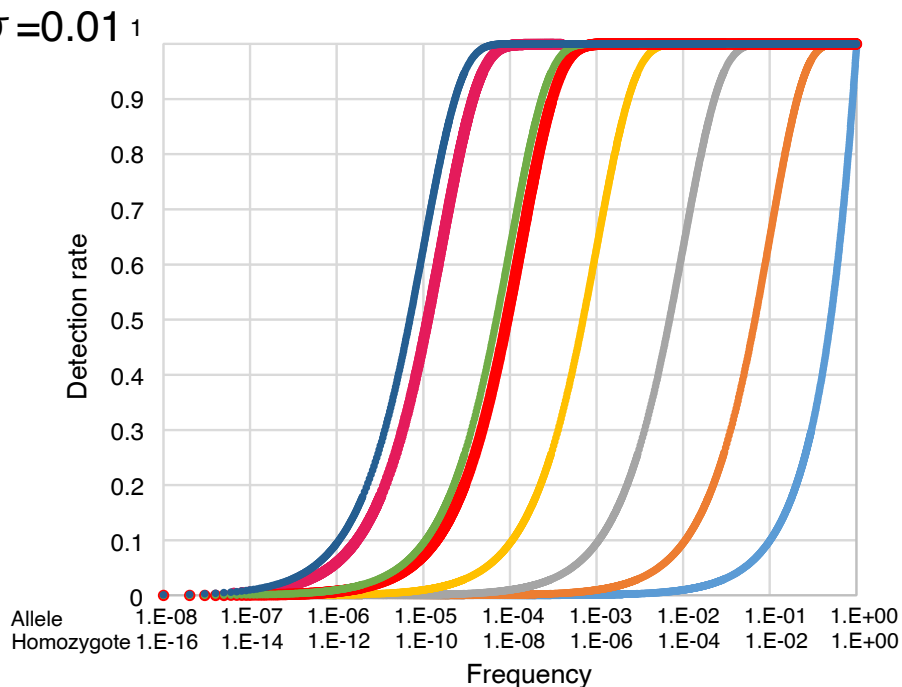

(c)  $\sigma=0.1$

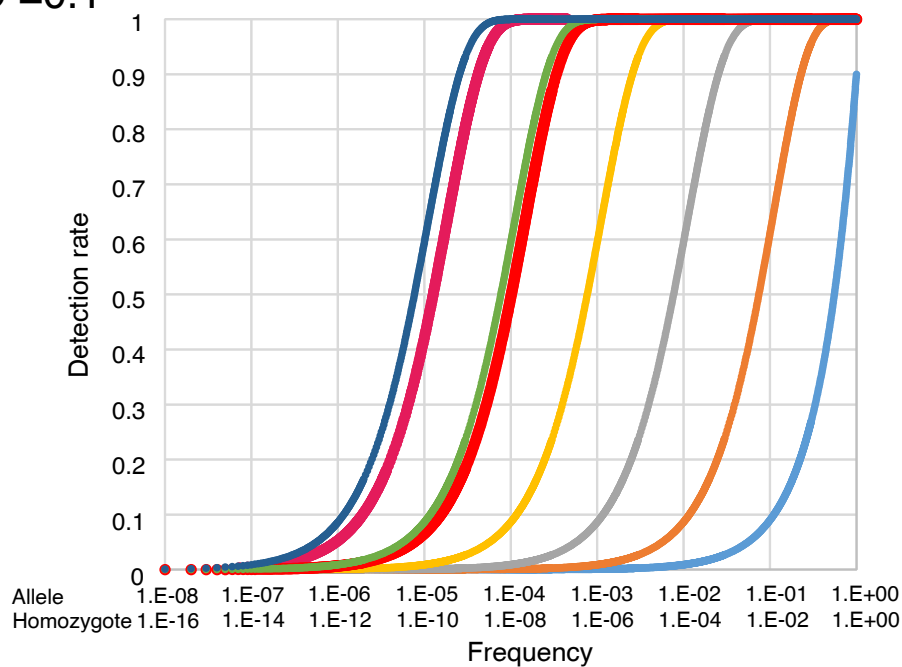

Supplement: S1 Fig — The theoretical mutation detection probability of high-penetrance genetic variants is calculated under the three condition (σ = 0; 0.01; 0.1) although the simulation under σ = 0.1 is unlikely situation. The simulation sample sizes range from 1 to 100,000. The y-axis corresponds to the detection rate of causative mutations. (PDF) [file pone.0155552.s001.pdf]
