# Supplementary material for: Global Carrier Rates of Rare Inherited Disorders Using Population Exome Sequences
Source: PLoS One. 2016 May 24;11(5):e0155552. doi: 10.1371/journal.pone.0155552 (PMC4878778; doi:10.1371/journal.pone.0155552)
Supplement: S1 Table — (PDF) [file pone.0155552.s002.pdf]

	Population	Total No.	%	male No.	female No.	male/female ratio %
1000 Genomes	AFR	246	22.5	115	131	46.7
	AMR	181	16.6	88	93	48.6
	ASN	286	26.2	144	142	50.3
	EUR	379	34.7	178	201	47.0
	ASW	61	5.59	24	37	39.3
	CEU	85	7.78	45	40	52.9
	CHB	97	8.88	44	53	45.4
	CHS	100	9.16	50	50	50.0
	CLM	60	5.49	29	31	48.3
	FIN	93	8.52	35	58	37.6
	GBR	89	8.15	41	48	46.1
	IBS	14	1.28	7	7	50.0
	JPT	89	8.15	50	39	56.2
	LWK	97	8.88	48	49	49.5
	MXL	66	6.04	31	35	47.0
	PUR	55	5.04	28	27	50.9
	TSI	98	8.97	50	48	51.0
	YRI	88	8.06	43	45	48.9
NHLBI	EA	4300	66.1	1872	2428	43.5
	AA	2203	33.9	571	1632	25.9
Total		7595		2968	4627	39.1
