# Supplementary material for: Global Carrier Rates of Rare Inherited Disorders Using Population Exome Sequences
Source: PLoS One. 2016 May 24;11(5):e0155552. doi: 10.1371/journal.pone.0155552 (PMC4878778; doi:10.1371/journal.pone.0155552)
Supplement: S2 Table — Target causative mutation lists analyzed in this study and representative reference lists. (PDF) [file pone.0155552.s003.pdf]

	Disease name	Gene name	Gene ID	Amino acid/nucleotide chang	dbSNP ID
1	Sickle cell anemia (MIM#603903)	*HBB* (hemoglobin, beta)	NM_000518.4	p.Glu7Val	rs77121243
2	Primary immunodeficiency (Mucocutaneous fungal infections) (MIM#613	*CLEC7A* (C-type lectin domain family 7, member	NM_197947.2	p.Tyr238*	rs16910526
3	PROP1-related combined pituitary hormone deficiency (CPHD) (MIM#26	*PROP1* (paired like homeodomain factor 1)	NM_006261.4	c.112_124del	
				c.149delGA	
				c.150delA	
				c.157delA	
				p.Arg73Cys	rs121917843
				p.Arg73His	rs121917842
				p.Gln83*	
				p.Phe88Ser	rs121917841
				c.296delGA	
				p.Arg99*	rs121917844
				p.Arg99Gln	rs137853100
				p.Leu102Cysfs*8	rs193922688
				c.310delC	
				p.Arg112*	
				c.343-11C >G	
				p.Phe117Ile	rs121917840
				p.Arg120Cys	rs121917839
				p.Arg125Trp	rs146918863
				c.467insT	
				p.Trp194*	rs121917845
4	Canavan disease (#271900)	*ASPA* (aspartoacylase)	NM_000049.2	c.32delT	
				p.Glu24Gly	rs104894551
				p.Arg71His	rs104894553
				c.433-2A>G	
				p.Cys152Arg	rs104894548
				p.Cys218*	rs104894549
				p.Tyr231*	rs12948217
				p.Asp249Val	rs104894552
				p.Glu285Ala	rs28940279
				c.876delAGAA	
				p.Ala305Glu	rs28940574
5	Pustular psoriasis (MIM no description)	*AP1S3* (adaptor-related protein complex 1, sigma 3	NM_001039569	p.Phe4Cys	rs116107386
				p.Gln17Lys	
				p.Thr22Ala	rs149183052
				p.Thr32Ile	rs78536455
				p.Arg33Trp	rs138292988
				p.Ile83Thr	rs202157374
6	Rod-cone dystrophy (RCD) (MIM#615780)	*PLK1S1* (polo-like kinase 1 substrate 1)	NM_018474.4	p.Glu18*	rs587777376
				p.Lys40Ilefs*14	rs587777377
				p.Arg76*	rs202210819
7	Primary autosomal recessive microcephaly 1 (MIM#251200)	*MCPH1* (microcephalin 1)	NM_024596.3	p.Ser25*	rs121434305
				p.Tyr27Arg	rs199422124
				p.Gln46*	
				p.His49Gln	
				p.Ser72Leu	rs387906961
				p.Trp75Arg	
				p.Ser101*	
				c.436+1G>T	
				p.Thr143Asnfs	rs199422125
				c.566Ains	
8	Seckel syndrome 5 (SCKL5) (MIM#613823)	*CEP152* (centrosomal protein 152kDa)	NM_001194998	p.Pro65Alafs*81	
				p.Lys667Arg	rs200879436
				p.Tyr678*	rs182018947
				p.Ile899Leufs*29	
				p.Val1404fs*2	
9	Pontocerebellar hypoplasia type 1B (PCH1B) (MIM#614678)	*EXOSC3* (exosome component 3)	NM_001002269	c.2T>C	
				p.Gly31Ala	rs387907196
				p.Pro52fs	
				p.Asp76fs	
				c.294_303del	
				p.Tyr109Asn	
				p.Asp132Ala	rs141138948
				p.Gly135Gln	
				p.Ala139Pro	rs387907195
				c.475-1269A>G	rs370087266
				p.Val80Phe	rs374550999
				p.Trp238Arg	
10	Miller syndrome (MIM#263750)	*DHODH* (dihydroorotate dehydrogenase)	NM_001361.4	p.Gly19Glu	rs267606765
				p.Glu52Gly	
				p.Arg135Cys	rs201230446
				p.Gly152Arg	rs267606766
				p.Arg199Cys	rs267606769
				p.Gly202Ala	
				p.Gly202Asp	rs267606767
				p.Leu204Profs*8	
				p.Arg244Trp	rs267606768
				p.The284Ile	
				p.Arg326*	
				p.Arg346Trp	rs201947120
				p.Ala357Thr	
				p.Asp392Gly	
11	Facial dysmorphism, lens dislocation, anterior-segment abnormalities, and spontaneous filtering blebs (FDLAB, or Traboulsi syndrome)	*ASPH* (aspartate beta-hydroxylase)	NM_004318.3	p.Asn618Glyfs*20	
				p.Arg735Trp	rs374385878
12	Carpenter syndrome (MIM#201000)	*RAB23* (RAB23, member RAS oncogene family)	NM_016277.4	p.Met12Lys	
				p.Arg28*	
				p.Tyr29*	
				p.Glu48fs*7	
				p.Val53fs*13	
				p.Tyr78fs*30	
				p.Tyr79del	
				p.Cys85Arg	
				p.Asn121fs*4	
				p.Glu137*	
				p.Leu145*	rs121908171
				c.482-1G>A	
13	Familial glucocorticoid deficiency (FGD) (MIM#614736)	*NNT* (nicotinamide nucleotide transhydrogenase)	NM_012343.3	c.1A>G	
				p.Ser22Profs*6	
				p.Ser193Asn	
				p.Tyr201Lysfs*1	
				p.Phe215Ser	
				p.Thr357Ala	
				p.His365Pro	
				p.His370*	
				p.Gln383*	
				p.Pro437Leu	
				p.Gln452Argfs*44	
				p.Ala533Val	
				p.Gln557*	

				p.Ile622Aspfs*1	
				p.Gly664Arg	rs371979800
				p.Gly678Arg	
				p.Thr689Leufs*320	
				p.Met880*	
				p.Leu977Pro	
				p.Ala1008Pro	
				p.Asn1009Lys	
14	childhood-onset dilated cardiomyopathy (MIM no description)	*RAF1* (Raf-1 proto-oncogene, serine/threonine kina	NM_002880.3	p.Ala237Thr	
				p.Arg254fs	
				p.Thr310Ala	
				p.Pro332Ala	
				p.Leu603Pro	
				p.His626Arg	
				p.Thr641Met	
15	Usher syndrome type 1J (USH1J) (#614869)	*CIB2* (calcium and integrin binding family member	NM_006383.2	p.Glu64Asp	rs145415848
				p.Phe91Ser	rs397515411
				p.Cys99Trp	rs370965183
				p.Ile123Thr	rs397515412
16	Aicardi-Goutières syndrome 6 (#615010)	*ADAR* (adenosine deaminase, RNA-specific)	NM_001111.4	p.Pro193Ala	rs145588689
				p.Lys359Argfs*14	rs398122898
				p.Phe535fs*	
				p.Arg544*	
				p.Ala870Thr	rs398122893
				p.Ile872Thr	rs398122897
				p.Arg892His	rs398122892
				p.Lys999Asn	
				p.Gly1007Arg	
				p.Tyr1112Phe	
				p.Asp1113His	
17	MEGDEL syndrome (MIM#614739)	*SERAC1* (serine active site containing 1)	NM_032861.3	c.128 +4A>G	
				p.Arg68*	
				p.Arg148*	rs387907236
				p.Ser156fsCys*16	
				p.Leu193Serfs*9	
				c.698-9TG>AGTGATA	
				c.1018delT	
				p.Gln390Profs*29	
				p.Gly401Asp	
				p.Gly404Glu	
				p.Trp438*	
				c.1403+1G>C	
				p.Leu479del	
				p.Ser498Thr	
				p.Gly536Ilefs*56	
				p.Ser543Phefs*44	
				c.1822_1828+10delTCAGCA	
				p.Gln642*	
18	severe dermatitis, multiple allergies and metabolic wasting (SAM syndrome) (MIM no description)	*DSG1* (desmoglein 1)	NM_001942.3	c.49–1G>A	
				p.Ala621Glnfs*3	
				p.Arg887*	
