# Supplementary material for: Global Carrier Rates of Rare Inherited Disorders Using Population Exome Sequences
Source: PLoS One. 2016 May 24;11(5):e0155552. doi: 10.1371/journal.pone.0155552 (PMC4878778; doi:10.1371/journal.pone.0155552)
Supplement: S3 Table — The P-value is calculated from pair-wise proportion tests of allele frequencies in European and African ancestries between the two different exome resources (1000 Genomes vs NHLBI). (PDF) [file pone.0155552.s004.pdf]

		1	2	3	4		5		
	Disease name	Sickle cell anemia	Primary immunodeficiency (Mucocutaneous fungal infection)	Pituitary hormone deficiency, combined 2 (CPHD2)	Canavan disease		Pustular psoriasis		
	OMIM entry	#603903	#613108	#262600	#271900				
	Gene name	*HBB*	*CLEC7A*	*PROP1*	*ASPA*		*AP1S3*		
	dbSNP	rs77121243	rs16910526	rs193922688	rs28940279	rs28940574	rs116107386	rs149183052	rs78536455
European origin	NHLBI EA	0.00023 (2/8596)	0.0790 (679/8600)	0.0011 (9/8254)	0.00023 (2/8600)	0.00023 (2/8600)	0.0128 (105/8232)	0.0013 (11/8188)	0.0069 (57/8280)
	1000 Genomes EUR	0 (0/758)	0.067 (51/758)	0 (0/758)	0 (0/758)	0 (0/758)	0.011 (8/758)	0.003 (2/758)	0.007 (5/758)
	p-value	1	0.28	0.757	1	1	0.726	0.397	1
African origin	NHLBI AA	0.0402 (177/4402)	0.0263 (116/4406)	0 (0/4264)	0.00023 (1/4406)	0 (0/4406)	0.0027 (10/3764)	0.00054 (2/3726)	0.00027 (1/3702)
	1000 Genomes AFR	0.091 (45/492)	0.014 (7/492)	0 (0/492)	0 (0/492)	0 (0/492)	0.004 (2/492)	0 (0/492)	0 (0/492)
	p-value	**4.04E-07**	0.14	1	1	1	0.919	1	1

			6	7	8		9		
	Disease name		Rod-cone dystrophy (RCD)	Primary autosomal recessive microcephaly 1 [illegible]	Seckel syndrome 5 (SCKL5)		Pontocerebellar hypoplasia type 1B (PCH1B)		
	OMIM entry		#615780	#251200	#613823		#614678		
	Gene name		*PLK1S1*	*MCPH1*	*CEP152*		*EXOSC3*		
	dbSNP	rs138292988	rs202210819	rs121434305	rs200879436	rs182018947	rs374550999	rs141138948	rs370087266
European origin	NHLBI EA	0.0116 (95/8174)	0.00061 (5/8180)	0.00012 (1/8222)	0.00012 (1/8230)	0.00024 (2/8366)	0.00012 (1/8586)	0.0013 (11/8600)	0.00012 (1/8600)
	1000 Genomes EUR	0.008 (6/758)	0 (0/758)	0 (0/758)	0 (0/758)	0 (0/758)	0 (0/758)	0 (0/758)	0 (0/758)
	p-value	0.457	1	1	1	1	1	0.432	1
African origin	NHLBI AA	0.0019 (7/3696)	0 (0/3660)	0 (0/3754)	0.0129 (49/3812)	0.0018 (7/3976)	0 (0/4384)	0 (0/4406)	0 (0/4406)
	1000 Genomes AFR	0 (0/492)	0 (0/492)	0 (0/492)	0.012 (6/492)	0.002 (1/492)	0 (0/492)	0 (0/492)	0 (0/492)
	p-value	0.704	1	1	1	1	1	1	1

		10			11	12	13	14	15
	Disease name	Miller syndrome			Facial dysmorphism, lens dislocation, anterior-segment abnormalities, and spontaneous filtering blebs (FDLAB, or Traboulsi syndrome)	Carpenter syndrome 1 (CRPT1)	Glucocorticoid deficiency 4 (GCCD4)	Aicardi-Goutières syndrome 6 (AGS6)	MEGDEL syndrome
	OMIM entry	#263750			#601552	#201000	#262600	#271900	#614739
	Gene name	*DHODH*			*ASPH*	*RAB23*	*NNT*	*ADAR*	*SERAC1*
	dbSNP	rs201230446	rs267606766	rs201947120	rs374385878	rs121908171	rs371979800	rs145588689	-
European origin	NHLBI EA	0.00061 (5/8232)	0.00012 (1/8302)	0.00024 (2/8362)	0.000116 (1/8600)	0.000233 (2/8590)	0.000116 (1/8600)	0.00372 (32/8600)	0.000121 (1/8254)
	1000 Genomes EUR	0 (0/758)	0 (0/758)	0 (0/758)	0 (0/758)	0.00264 (2/758)	0 (0/758)	0.00132 (1/758)	0 (0/758)
	p-value	1	1	1	1	**0.031**	1	0.45	1
African origin	NHLBI AA	0 (0/3790)	0 (0/3878)	0 (0/4052)	0 (0/4406)	0.00454 (2/4404)	0 (0/4406)	0.00204 (9/4406)	0 (0/4264)
	1000 Genomes AFR	0 (0/492)	0 (0/492)	0 (0/492)	0 (0/492)	0 (0/492)	0 (0/492)	0 (0/492)	0 (0/492)
	p-value	1	1	1	1	1	1	0.65	1
